# Identification of two transcription factors that work coordinately to regulate early development in *Entamoeba*

**DOI:** 10.1128/mbio.02250-24

**Published:** 2024-11-14

**Authors:** Daniela Lozano-Amado, Upinder Singh

**Affiliations:** 1 Division of Infectious Diseases, Stanford University School of Medicine, Palo Alto, California, USA; 2 Department of Microbiology and Immunology, Stanford University School of Medicine, Palo Alto, California, USA; University of Wisconsin-Madison, Madison, Wisconsin, USA

**Keywords:** *Entamoeba invadens*, transcriptional regulators, encystation

## Abstract

The protozoan parasite *Entamoeba* has a life cycle that switches between infective cysts and invasive trophozoites. Encystation, a crucial process in parasite biology, is controlled by different mechanisms including transcriptional control. We identified two nuclear proteins in *Entamoeba invadens*, EIN_066100 and EIN_085620, that regulate parasite development by binding to a DNA motif (TCACTTTC) in the promoter regions of genes upregulated in the first 8 h of stage conversion. Overexpression of EIN_066100, a homolog of MAK16 protein, resulted in reduced amoebic proliferation without affecting encystation efficiency. Overexpression of EIN_085620, a protein with an RNA-recognition motif (RRM), led to increased encystation efficiency. Glutathione S-transferase (GST) pull down assays revealed that EIN_066100 interacts with EIN_085620 both *in vivo* and *in vitro,* and this interaction is mediated by the EIN_085620 RRM domain. By evaluating truncated proteins with deletions at either the N-terminal or C-terminal regions of EIN_066100, we elucidated the importance of its N-terminal region in proper protein localization, proliferation, encystation, and interaction with EIN_085620. Taken together, these results indicate a coordinated role of EIN_066100 and EIN_085620 in regulating *Entamoeba* development. This work sheds light on the molecular mechanisms in the earliest stages of *Entamoeba* encystation.

**IMPORTANCE:**

An important biological process in the biology of *Entamoeba* is stage conversion, which plays a crucial role in disease propagation, facilitating parasite survival outside the host and spreading to new hosts. Multiple mechanisms contribute to controlling the expression of amebic stage-specific genes such as epigenetic and transcriptional control. Identification of early transcriptional control regulators is crucial to understanding the initiation of the encystation cascade. We identified two nuclear proteins, EIN_066100 and EIN_085620, involved in the proliferation and developmental regulation of *E. invadens*. These proteins work by direct binding to each other and mediating encystation efficiency. Study of new regulators involved in *Entamoeba* development represents an important advance in a critical aspect of parasite biology.

## INTRODUCTION

*Entamoeba histolytica* is a unicellular parasitic protozoan that causes amebiasis in humans. This intestinal infection affects millions of people worldwide, and the mortality due to this parasitic disease is approximately 55,000 people annually (1). *E. histolytica* has a life cycle that alternates between two stages, consisting of a dormant and infective cyst and a motile and invasive trophozoite. Encystation, the process in which trophozoites transform into mature cysts, is a crucial point for survival outside the host and spreading infection (2). During this process, the parasite undergoes morphological changes: cellular aggregation occurs within the first 8 to 12 h, rounded cells and immature cysts form at 24 h, cyst maturation continues at 48 h, and mature quadrinucleated cyst is formed with a robust cyst wall at 72 h (3–5).

Although a reproducible method for inducing encystation in the human pathogen *E. histolytica* has been recently described (6), challenges remain in consistently obtaining mature cysts *in vitro*. Therefore, the reptilian parasite *Entamoeba invadens* continues to be widely used as a model to elucidate encystation-associated processes (7, 8). Several mechanisms involved in encystation have been studied, such as metabolic and signaling pathways (9, 10), epigenetic factors (11), and more recently transcriptional networks (12–15). To date, four transcription factors responsible for regulating amebic development have been identified. These include a *E. histolytica* Myb protein (EhMyb-dr), which belongs to the SHAQKY family and binds to a CCCCCC promoter motif (12), a novel encystation regulatory motif-binding protein (ERM-BP) that is regulated by NAD+ and binds to the CAACAAA DNA motif in the promoters of cyst-specific genes at 24 h (13); the nuclear factor complex (NF-Y), which binds to a conserved CCAAT motif and appears at a later time point of encystation (14); and the TALE homeobox protein in *E. histolytica* (EhHbox) and *E. invadens* (EiHbox1) that is upregulated during the early hours of encystation and binds TGACAG and TGATTGAT motifs (15). All these transcription factors contribute to controlling the expression of amoebic stage-specific genes at different time points, particularly at 24 h and 48 h post-encystation. However, identifying the earliest transcriptional control regulators upstream of previously identified transcription factors would help understand how the encystation cascade is initiated.

The RNA-recognition motifs (RRMs) are common and well-studied RNA-binding domains (16) that can bind different molecules, mainly single-stranded RNA and DNA, as well as proteins (17). Proteins containing the RRM domain are involved in post-transcriptional processes such as pre-processing of mRNAs, alternative splicing, stability, editing, and export of mRNAs to regulate gene expression (18). Although not all RRMs can bind DNA, proteins that bind both DNA and RNA regulate many cellular processes, including transcription, translation, gene silencing, microRNA biogenesis, and telomere maintenance (19).

In this work, we identified two proteins in *E. invadens*, EIN_066100 and EIN_085620, which bind to DNA motif (TCACTTTC) found in the promoters of genes upregulated at 8 h post-encystation. Both EIN_066100 and EIN_085620 are upregulated at early encystation and have a nuclear localization in trophozoites and cysts. Overexpression of EIN_066100 did not affect encystation efficiency, whereas overexpression of EIN_085620 increased encystation. Glutathione S-transferase (GST) pull down assays demonstrate that EIN_066100 interacts with EIN_085620 *in vivo* and *in vitro,* and this interaction is mediated by the EIN_085620 RRM domain. The functions of EIN_066100 were evaluated by overexpressing mutant versions of the protein in *Entamoeba* parasites and demonstrated that deletion of the N-terminal region of EIN_066100 led to mis-localization of the mutant protein in both trophozoites and cysts and significantly reduced proliferation and encystation efficiency. Furthermore, deletion of the N-terminal region disrupted the interaction with EIN_085620. Overall, our results indicate a coordinated role of EIN_066100 and EIN_085620 in regulating the early stages of developmental control. The discovery of these novel transcriptional regulators in *Entamoeba* development enhances our understanding of the molecular basis of amebic stage conversion, a crucial aspect of parasite biology.

## RESULTS

### Identification of conserved DNA motifs in the promoters of genes upregulated early (8 h post-encystation) in *E. invadens*

In order to identify the transcriptional network involved in early time points of encystation; we utilized previously generated RNA-seq data of different time points of *E. invadens* encystation (20). Analysis of the transcriptome at 8 h of stage conversion revealed that 502 genes are upregulated 3-fold (with *P*-value < 0.05) compared with trophozoites (Table S1). Promoter sequences of this subset of genes were bioinformatically analyzed with MEME and FIMO to identify conserved DNA motifs. Four motifs, M6, M9, M11, and M15, were significantly enriched among the promoters (Fig. S1).

### M9 motif is a potential DNA-binding sequence and binds nuclear proteins in cysts

**Fig 1:**
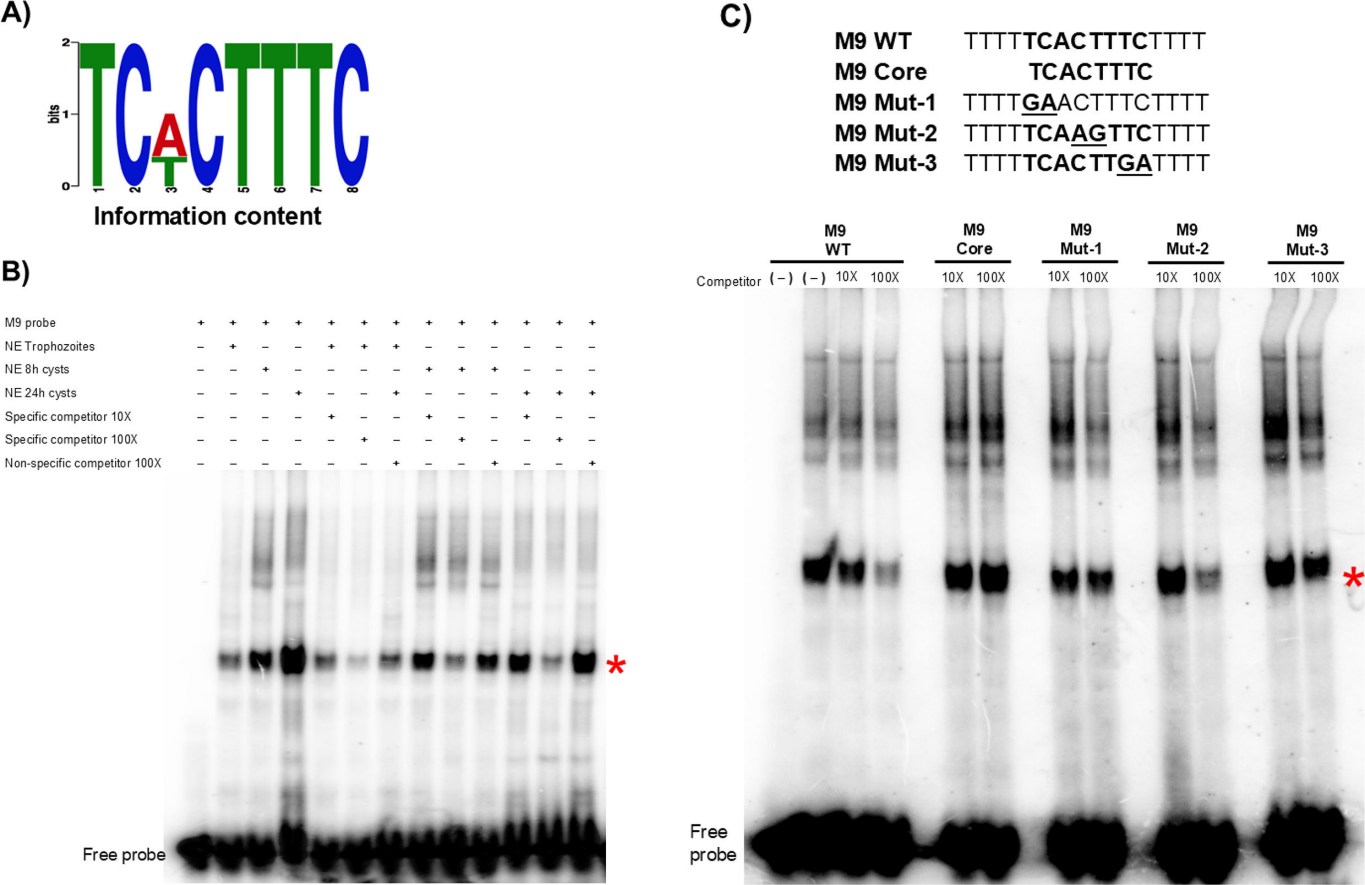
M9 motif is a potential DNA-binding sequence that is strongly recognized by cyst nuclear proteins. (**A**) Sequence logo of M9 motif, which is enriched in promoters of genes upregulated at 8 h encystation. The eight-nucleotide motif information content is shown. (**B**) Representative EMSA results in the presence of nuclear extracts from trophozoites, 8 h cysts and 24 h cysts. A radiolabeled M9 probe was used in each reaction. Unlabeled M9 probe at 10× and 100× was used as a specific competitor; a non-specific ERM-BP cold competitor was used at 100×. The red asterisk indicates the major specific band in the gel shift assay; a free probe is at the bottom. (**C**) Sequences of three mutants (Mut-1, 2, and 3) were generated by changing the conserved TC nucleotides to GA (underlined) and M9 core. Competition assays using 8 h cysts nuclear extracts were performed using 10× and 100× of a cold competitor against radiolabeled M9 WT probe. The red asterisk indicates the major band that exhibits specific binding.

We screened all four motifs by electrophoretic mobility shift assay (EMSA) using nuclear proteins from trophozoites, and parasites at 8 h and 24 h post-encystation. Of the four motifs, only M9 (TCACTTTC) (Fig. 1A) showed binding to nuclear extracts from trophozoites with binding increasing during early encystation (8 h and 24 h) (Fig. 1B). The specificity of M9 motif was tested by competition assays using nuclear extracts and specific and non-specific competitors. Competition by specific but not by non-specific competitors demonstrated that the DNA complex is specific for the M9 motif (Fig. 1B). M9 motif is found in the promoters of 85 genes of the 502 genes upregulated at 8 h, suggesting that it controls a specific subset of early developmentally regulated genes.

To identify the critical residues in the M9 motif, EMSA using three mutant probes (Mut-1, Mut-2, and Mut-3) was performed (Fig. 1C). Mut-1 (GAACTTTC) and Mut-3 (TCACTTGA) did not show the ability to compete at 10× and 100×, suggesting that the TC nucleotides at positions one, two, seven, and eight in the M9 motif are important for binding to encysting nuclear protein(s). Furthermore, our results indicate that M9 core (consisting of the M9 sequence without the linker) exhibited a limited ability to compete, indicating that this short motif may not be highly effective in binding to nuclear proteins, and flanking sequences are needed for establishing a physical interaction, as observed by earlier investigations (13, 21).

### Identification of EIN_066100 and EIN_085620 as M9-binding proteins

To identify the M9-binding protein(s), EMSA shifted bands using 8 h cysts nuclear extracts were excised from a polyacrylamide gel and analyzed by liquid chromatography–mass spectrometry (LC-MS). The protein content of the positive shifted band (M9 WT) was compared with the same gel mobilities in the negative controls (M9 Core and ERM-BP motif) in two biologically independent experiments (Table S2; Fig. S2). Our analysis showed that only four proteins were exclusive in M9-WT compared with negative controls. These four proteins include a putative maintenance of killer 16 protein (EIN_066100), a putative pre-rRNA-processing protein esf2 (EIN_085620), a putative glycoprotein FP21 precursor (EIN_267870), and a hypothetical protein (EIN_399700). All these proteins are conserved among different *Entamoeba* species and were cloned and transfected into *E. invadens* trophozoites. EIN_066100, EIN_085620, and EIN_267870 proteins were Myc-tagged successfully expressed in the parasite (data not shown), and only two showed nuclear localization. Based on these results, we identified EIN_066100 and EIN_085620 as our two potential candidates for M9-binding proteins and selected them for further studies.

### Characterization of EIN_066100 and EIN_085620

**Fig 2:**
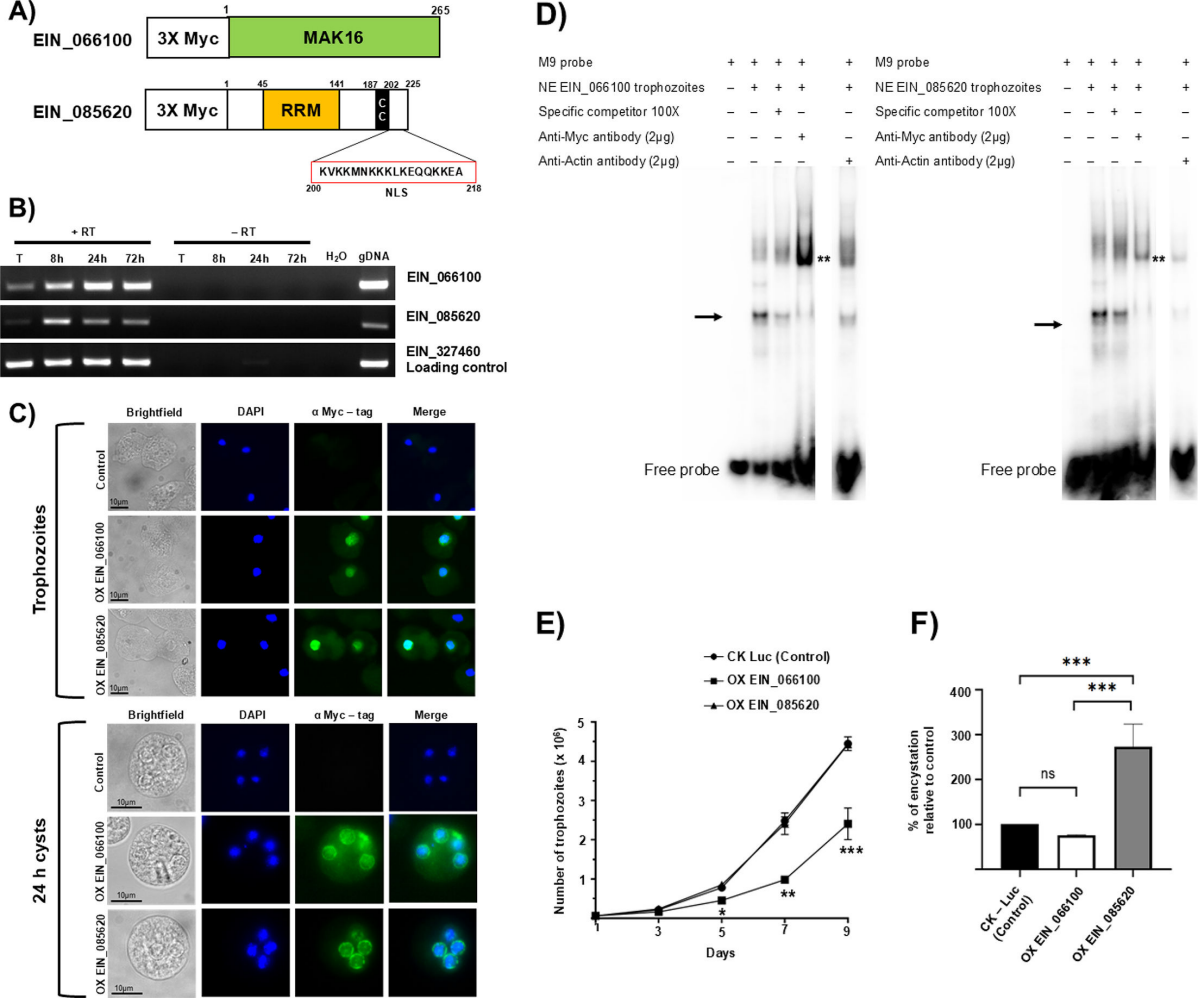
Myc-tagged EIN_066100 and EIN_085620 are nuclear proteins and have an effect on amoebic proliferation and encystation. (**A**) Schematic representation of EIN_066100 and EIN_085620. EIN_066100 is a homolog of Mak16 protein (green). EIN_085620 is a homolog of Esf2 and has an RRM domain (orange), a coiled-coil domain (CC) domain (black), and a putative NLS (red). The length of proteins is drawn to scale and the amino acids corresponding to each domain are indicated. (**B**) RT-PCR to detect the expression level of EIN_066100 and EIN_085620 transcripts at different time points of encystation. EIN_327460, a gene that does not change expression during development, was a loading control. For each sample, a reaction was performed with reverse transcriptase (+RT), without reverse transcriptase (–RT), and no template as a negative control. (**C**) Immunostaining with anti-Myc antibody (green) in trophozoites (top) and 24 h cysts (bottom) was performed in IP1 (untransfected control), OX EIN_066100, and OX EIN_085620 cell lines. Nuclei are stained with DAPI (Blue). (**D**) Gel super-shift assay using radiolabeled M9 probe, nuclear extracts from Myc-tagged OX EIN_066100 and OX EIN_085620 with rabbit polyclonal anti-Myc and α-actin antibodies at 2 µg. The black arrow indicates major bands that exhibit binding to M9. ** indicates super-shifted bands most likely due to complex formation with anti-Myc antibody. (**E**) Growth kinetics for the CK-Luc, OX EIN_066100, and OX EIN_085620 cell lines. A two-way ANOVA was used to detect significant differences between groups across all time points. A significant reduction in the growth rate of the OX EIN_066100 cell line on days 5, 7, and 9 compared with the CK-Luc cell line was observed. (F) Percentage of encystation relative to control in CK-Luc, OX EIN_066100, and OX EIN_085620 cell lines after 72 h of encystation. A one-way ANOVA was used to detect significant differences between groups. Graph shows mean ± S.D. (*n* = 3), and the significance values were assigned by the asterisks: *P < 0.05, **P < 0.01, ***P < 0.001.

In order to elucidate the role of EIN_066100 and EIN_085620 in *Entamoeba*, we analyzed the protein sequences to identify conserved domains. Our analysis using different databases such as InterPro, Pfam, and SMART showed that EIN_066100 is a homolog of MAK16 protein, a nucleolar protein previously studied in *Saccharomyces cerevisae* (22, 23) and *Schistosoma mansoni* (24, 25). EIN_085620 is a homolog of the Esf2 protein, previously studied in yeast (26), and the mABT1 protein is identified in mice (27). It contains an RNA recognition motif (RRM), a putative NLS sequence, and a short coiled-coil domain (Fig. 2A). Homologs of these proteins were identified in *E. histolytica* (EHI_198690A and EHI_174110A) (Fig. S3). EIN_066100 shows a 69% identity with EHI_198690A, whereas EIN_085620 shows about 40% identity with EHI_174110A. Interestingly, the RNA recognition motif (RRM) domain found in EIN_085620 has also been identified in *E. histolytica*. This high sequence similarity and conservation of functional domains in *E. histolytica* and *E. invadens* support the functional relevance of these proteins in both species.

To define whether these genes have a stage-specific expression, we performed semiquantitative reverse transcriptase PCR (RT-PCR) in *E. invadens* trophozoites and cysts at different time points of encystation (8, 24, and 72 h) (Fig. 2B). In trophozoites, both EIN_066100 and EIN_085620 were expressed at relatively lower levels, with EIN_066100 exhibiting slightly higher expression. Interestingly, both genes showed a sequential upregulation during the early stages of encystation. Our RT-PCR results are consistent with the previously published RNA-seq data (20), suggesting that the earliest transcription factors may be initially expressed during the trophozoite stage and increase their expression when encystation cascade is activated.

To determine the subcellular localization, immunofluorescence assays were performed on Myc-EIN_066100 and Myc-EIN_085620 overexpressing cell lines using an anti-Myc antibody. Both proteins showed a weak cytoplasmic signal and strong nuclear staining in trophozoites, whereas in cysts, the signal was predominantly observed around the nuclei, suggesting a perinuclear localization (Fig. 2C). Nuclear localization of EIN_066100 and EIN_085620 supports the hypothesis that these proteins can act as transcription factors.

To demonstrate whether EIN_066100 and EIN_085620 are part of a protein complex that binds to the M9 motif, we performed gel super-shift assays using nuclear extract from Myc-tagged EIN_066100 and EIN_085620 overexpressing trophozoites, anti-Myc antibody, and anti-actin antibody (Fig. 2D). EMSA with anti-Myc antibody resulted in a super-shift band (**) for EIN_066100, which was stronger compared with the band for EIN_085620. The actin antibody did not result in a super-shift. Taken together, the data suggest that EIN_066100 and EIN_085620 might be part of a nuclear complex that binds the M9 sequence.

To better understand EIN_066100 and EIN_085620 roles in amebic biology, we monitored the growth kinetics for overexpressed cell lines (Fig. 2E). We did not observe significant changes in the growth rate of EIN_085620 compared with the CK-Luc control. However, EIN_066100 overexpression demonstrated a significant reduction in cell division at days 5, 7, and 9, suggesting that EIN_066100 may play a role, either directly or indirectly in regulating amoeba proliferation. Alternatively, it is possible that the overexpression of EIN_066100 activates signaling pathways linked to the initiation of encystation, which could lead to a slowdown in cell growth.

In order to determine whether overexpression of EIN_066100 and EIN_085620 has an effect on encystation, we induced encystation in all three cell lines, keeping the initial cell number constant, and counted the number of cysts after 72 h to calculate the percentage of encystation relative to the CK-Luc cell line control (Fig. 2F). Although EIN_066100 had an effect on the proliferation rate in trophozoites, it did not cause a significant change in encystation compared with the control. However, EIN_085620 overexpression increased the percentage of encystation by up to 2.5-fold, suggesting that EIN_085620 plays a positive role in regulating encystation.

### EIN_066100 and EIN_085620 interact *in vitro* and *in vivo*

**Fig 3:**
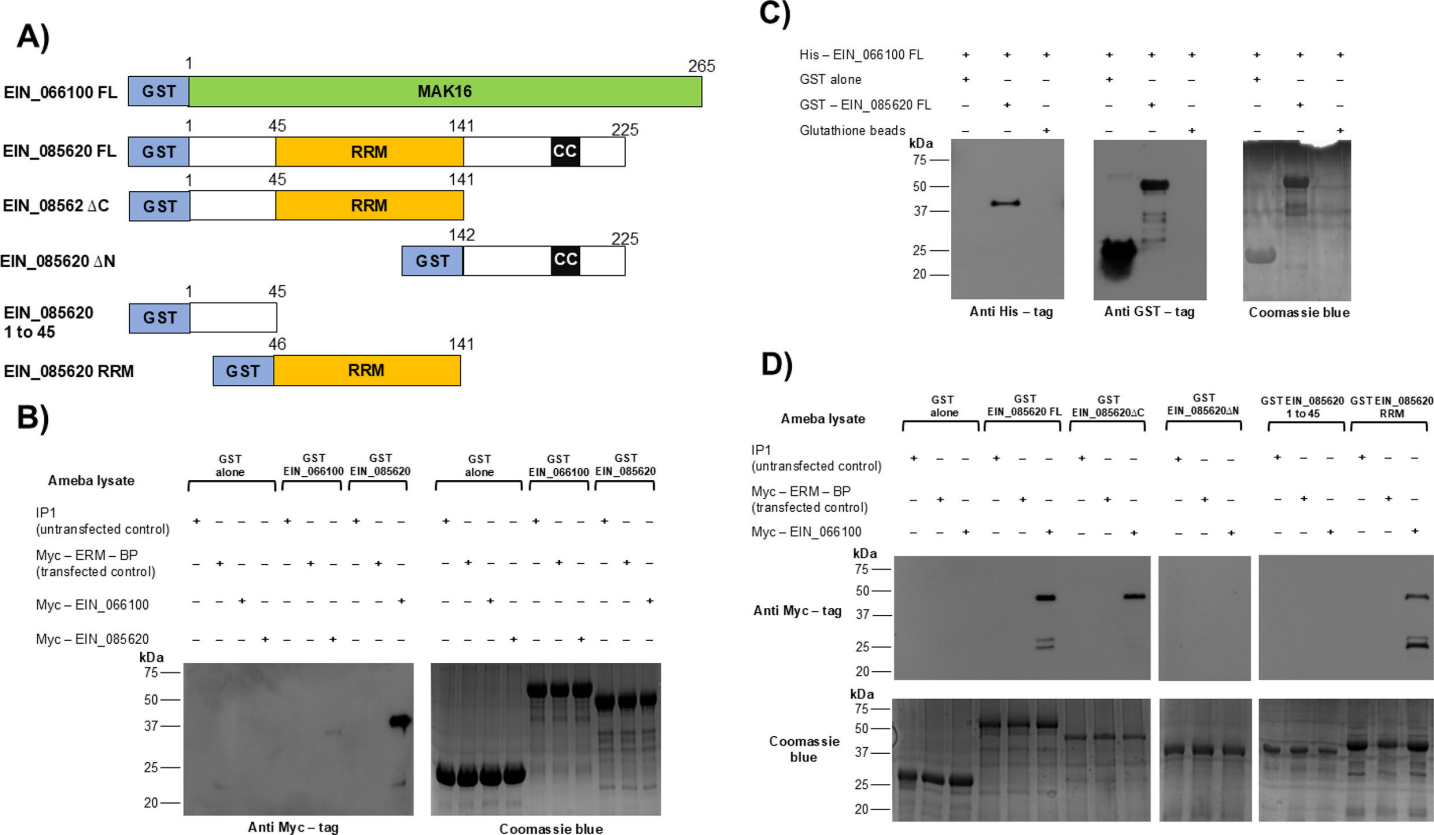
EIN_066100 and EIN_085620 proteins interact *in vitro* and *in vivo* via the EIN_085620 RRM domain. (**A**) Schematic representation of the EIN_066100 FL, EIN_085620 FL, and deletion mutants (EIN_085620∆C, EIN_085620∆N, EIN_085620 1 to 45 and EIN_085620 RRM). The amino acid positions relevant to the deletions made are indicated. (**B**) GST pull down assay shows *in vivo* interaction between Myc-EIN_066100 and GST-EIN_085620 FL. GST-tagged from the bacterial lysate were immobilized by glutathione beads and used as “bait.” The beads were incubated with amoebic lysates containing “prey” Myc-tagged EIN_066100, Myc-tagged EIN_085620, and Myc-tagged ERM-BP. After incubation and washing, bound complexes were eluted and analyzed by western blot using anti-Myc antibody. Weak Myc-tagged EIN_085620 signal (expected size 35 kDa) was observed in the bait sample lane of GST-EIN_066100; however, a noticeable Myc-tagged EIN_066100 signal (expected size 37 kDa) in lane sample containing GST-EIN_085620 “bait” was observed. No signal corresponding to Myc-tagged proteins was observed in the GST alone, used as a negative control. (C) Physical interaction *in vitro* was confirmed by GST pull down. GST-tagged EIN_085620 was used as “bait” and incubated with “prey” purified His-EIN_066100. Bound complex was eluted and analyzed by western blotting using anti-His-tag antibody and anti-GST antibody. His-EIN_066100 FL signal was observed (expected size 37 kDa) exclusively when was incubated with GST-EIN_085620 (expected size 52 kDa). The glutathione beads and GST alone do not pull the His-EIN_066100 protein. (**D**) GST pull down was performed with GST-tagged EIN_085620 FL, EIN_085620∆C, EIN_085620∆N, EIN_085620 1 to 45, and EIN_085620 RRM proteins incubated with amoebic lysates from Myc-tagged EIN_066100 and tagged ERM-BP. After interaction and washing, bound complexes were eluted and analyzed by western blot using anti-Myc antibody. Myc-tagged EIN_0666100 interacts with GST-tagged EIN_085620 FL as was previously observed. Deletion of C-terminal region (GST-EIN_085620∆C) does not affect the interaction with Myc-tagged EIN_066100; however, deletion of N-terminal region (GST-EIN_085620∆N) fails to interact with Myc-tagged EIN_066100. Mapping of EIN_085620 N-terminal region indicated that GST-EIN_085620 1 to 45 does not interact with Myc-tagged EIN_066100, and GST-EIN_085620 RRM is able to interact with Myc-tagged EIN_066100. No signal corresponding to Myc-tagged EIN_066100 was observed in GST alone used as a negative control. An extra negative control, Myc-tagged ERM-BP, showed no reactivity with all GST proteins.

The identification by LC-MS and nuclear localization of EIN_066100 and EIN_085620 suggest that these proteins might function together. To demonstrate a possible interaction between EIN_066100 and EIN_085620, we expressed these full-length proteins in *E. coli* with N-terminal GST and histidine (His) tags. All proteins showed an expression by sodium dodecyl sulfate-polyacrylamide gel electrophoresis (SDS-PAGE) at their expected sizes upon induction with isopropyl-β-D-thiogalactoside (IPTG), and we were able to purify both GST-tagged and His-tagged EIN_066100 and EIN_085620 (Fig. S4). To test a potential interaction between EIN_066100 and EIN_085620, a GST pulldown assay was performed. In this experiment, GST-tagged full-length EIN_066100 and GST-tagged full-length EIN_085620 were used as bait proteins to pull down Myc-tagged EIN_085620 and Myc-tagged EIN_085620 (Fig. 3B). GST-tagged EIN_066100 and GST-tagged EIN_085620 proteins were immobilized using glutathione-agarose beads. These beads were incubated with whole-cell lysates obtained from different amoeba cell lines, including Myc-tagged EIN_066100 and Myc-tagged EIN_085620. Bound proteins were eluted from beads and resolved by SDS-PAGE. Eluted samples were analyzed by Coomassie staining, which showed prominent bait proteins for each sample at their expected sizes (Fig. 3C, bottom). To check if Myc-tagged prey proteins were present, we probed the eluted samples by western blotting using anti-Myc-tag antibody. We observed a weak Myc-tagged EIN_085620 signal in the bait sample lane of GST-EIN_066100; however, a strong Myc-tagged EIN_066100 signal was observed in the lane of GST-EIN_085620, indicating that GST-EIN_085620 was able to interact *in vivo* with EIN_066100. Myc-tagged proteins were not observed in the GST alone, and GST-EIN_066100 and GST-EIN_085620 did not interact with other Myc-tagged proteins such as ERM-BP (EIN_083100), which was used as a negative control.

To substantiate the *in vivo* interaction, we checked whether EIN_066100 and EIN_085620 directly interact *in vitro*. GST-tagged EIN_085620 was used as “bait” protein and incubated with “prey” His-EIN_06. Bound proteins were resolved by SDS–PAGE and analyzed by western blotting using anti-His-tag antibody and anti-GST antibody (Fig. 3C). The results show that His-EIN_066100 was specifically pulled down when incubated with GST-tagged EIN_085620, indicating an *in vitro* interaction between them. We included GST alone and glutathione agarose beads as negative controls. Western blotting for anti-GST was done to show that GST proteins used as bait were in the pulled samples. Thus, we have confirmed the *in vivo* and *in vitro* interaction between full-length EIN_066100 and EIN_085620.

### EIN_085620 RRM domain is responsible for the interaction with EIN_066100

Bioinformatics searches for conserved protein motifs in EIN_085620 indicated that it has an RRM domain (45–141 aa) and a coiled-coil domain (187–202 aa) in their C-terminal region (Fig. 3A). In other systems like yeast, it has been demonstrated that C-terminal region of Esf2 is required for binding to Dbp8 and for stimulating Dbp8 ATPase activity (26). In mice, the region in mABT1 comprising residues 34–102 is necessary for binding to TBP (27).

To examine which regions of EIN_085620 are responsible for the interaction with EIN_066100, a series of GST-tagged EIN_085620 deletion mutants were generated (Fig. 3A), and their ability to interact with EIN_066100 was assayed in a directed GST pull down with whole-cell amoebic lysates (Fig. 3D left panel). Our data show that the C-terminal deletion mutant EIN_085620∆C did not affect the interaction with EIN_066100. In contrast, the N-terminal deletion mutant EIN_085620∆N failed to interact with Myc-tagged EIN_066100, suggesting that amino acids 1–141 are required for the interaction. Then, we focused on the mapping of EIN_085620 N-terminal region and generated two GST-tagged proteins: one of them corresponding to the first 45 amino acids and the second one corresponding to the RRM domain (Fig. 3D right panel). Our GST pull down results show that only GST-EIN_085620 RRM domain was able to interact with Myc-tagged EIN_066100, indicating that interaction of EIN_085620 with EIN_066100 occurs through EIN_085620 RRM domain.

### The N-terminal region of EIN_066100 is important for nuclear localization

**Fig 4:**
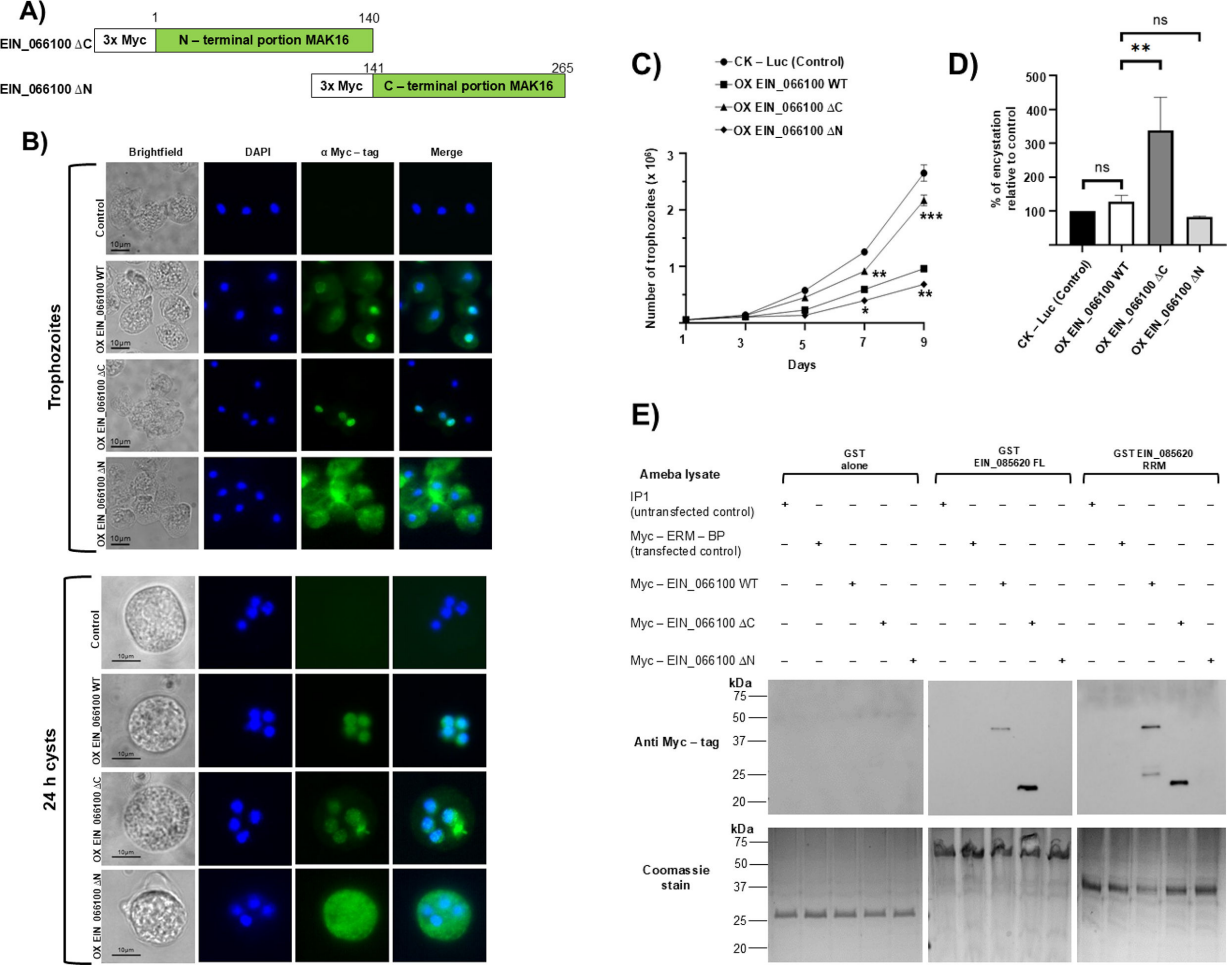
Expression of EIN_066100 mutants affects subcellular localization and rates of *Entamoeba* proliferation and encystation. (**A**) Schematic representation of EIN_066100∆C and EIN_066100∆N. The amino acid positions relevant to the deletions made are indicated. (**B**) Immunostaining with anti-Myc antibody (green) in trophozoites (top) and 24 h cysts (bottom) was performed in control, OX EIN066100 WT, OX EIN_066100∆C, and OX EIN_066100∆N cell lines. Nucleus is stained with DAPI (blue). (**C**) Growth kinetics for the CK-Luc, OX EIN_066100 WT, OX EIN_066100∆C, and OX EIN_066100∆N cell lines. A two-way ANOVA was used to detect significant differences between groups across all time points. A significant increase in the growth rate was observed in the OX EIN_066100∆C cell line, whereas OX EIN_066100∆N cell line showed a reduction in the growth rate compared with OX EIN_066100 WT. (**D**) Percentage of encystation relative to control in CK-Luc, OX EIN_066100 WT, OX EIN_066100∆C, and EIN_066100∆N cell lines after 72 h of encystation. A one-way ANOVA was used to detect significant differences between groups. Graphs show mean ± S.D. (*n* = 3), and the significance values were assigned by the asterisks: *P < 0.05, **P < 0.01, ***P < 0.001. (**E**) GST pull down was performed with GST-tagged EIN_085620 FL and EIN_085620 RRM proteins incubated with amoebic lysates from Myc-tagged EIN_066100 WT, EIN_066100∆C, EIN_066100∆N, and ERM-BP. After interaction and washing, bound complexes were eluted and analyzed by western blot using anti-Myc antibody. Myc-tagged EIN_0666100 WT interacts with GST-tagged EIN_085620 FL and GST-tagged EIN_085620 RRM as was previously observed. Myc-tagged EIN_066100∆C interacts with both GST-tagged proteins; however, Myc-tagged EIN_066100∆N fails to interact with GST-tagged proteins, indicating that the N-terminal region of EIN_066100 is needed to mediate the interaction with EIN_085620. No signal corresponding to Myc-tagged EIN_066100 WT, EIN_066100∆C, and EIN_066100∆N was observed in GST alone used as a negative control. An extra negative control Myc-tagged ERM-BP showed no reactivity with all GST proteins.

In order to gain insights into the role of EIN_066100 and EIN_085620 in *Entamoeba* differentiation, our next strategy was genetic downregulation. Despite multiple attempts using previously reported genetic tools (28), we were unsuccessful in downregulation of our genes of interest. As an alternative to evaluate the gain and loss of function of these proteins, we proceeded to generate EIN_066100 and EIN_085620 mutant parasites by deleting either the C-terminal or N-terminal regions (Fig. 4A) and assessing phenotypic outcomes. Although all the constructions were generated in an *E. invadens* expression vector under CK-promoter used to express the WT proteins, only EIN_066100∆C and EIN_066100∆N mutants were successfully expressed into the parasites. We evaluated the intracellular distribution of both EIN_066100 mutants in trophozoites and 24 h cysts by immunostaining with anti-Myc antibody. Overexpression of EIN_066100∆C mutant showed nuclear localization in both stages similar to wild-type EIN_066100. However, the mutant EIN_066100∆N was enriched in the cytoplasm in both trophozoites and cysts (Fig. 4B). These results suggest that the N-terminal region of EIN_066100 is important in determining its nuclear localization.

### EIN_066100 mutants affected the proliferation and encystation processes

To elucidate the functional roles of the identified protein domains, we determined whether overexpression of the deletion mutant proteins impacted amoeba proliferation. Consistent with our previous observations, EIN_066100 WT showed a significant reduction compared with CK-Luc control. Our results indicate that EIN_066100∆C mutant caused an increase in amoeba numbers compared with EIN_066100 WT on days 7 and 9. In contrast, expression of EIN_066100∆N mutant resulted in a significant growth defect at days 5, 7, and 9 compared with EIN_066100 WT (Fig. 4C). These results highlight the distinct regulatory roles played by the N-terminal and C-terminal domains of the EIN_066100 protein in modulating amoeba growth.

Furthermore, we assessed the effect of EIN_066100 mutants in encystation by counting the number of cysts after 72 h to calculate the percentage of encystation relative to the EIN_066100 WT cell line (Fig. 4D). Our findings indicate that proliferation rates might have influence of encystation. Compared with the EIN_066100 WT, the EIN_066100∆C mutant exhibited a significant increase in encystation, whereas the EIN_066100∆N mutant showed a modest but statistically significant reduction in encystation compared with the EIN_066100 WT.

Considering the phenotypic effects observed, our data indicate that EIN_066100∆N mutant acted as a dominant negative protein, whereas the EIN_066100∆C mutant exhibited a gain-of-function role, promoting both proliferation and encystation of *Entamoeba*.

### The N-terminal region of EIN_066100 is crucial for its interaction with EIN_085620

Our previous GST pull down experiments showed that EIN_066100 interacts with EIN_085620 both *in vivo* and *in vitro*. To investigate whether EIN_066100 mutants can also interact with EIN_085620, we conducted GST pull down assays with whole-cell amoebic lysates from EIN_066100∆C and EIN_066100∆N mutants and GST-tagged EIN_085620-FL and GST-tagged EIN_085620-RRM proteins (Fig. 4E). Our data show that the Myc-tagged EIN_066100∆C mutant was able to interact with both GST-EIN_085620-FL and GST-EIN_085620-RRM proteins, similar to the wild-type EIN_066100. In contrast, the Myc-tagged EIN_066100∆N mutant failed to interact with either GST-tagged EIN_085620 FL or GST-EIN_085620-RRM proteins. These findings suggest that in addition to its importance for nuclear localization, the N-terminal region of EIN_066100 is also crucial for mediating the interaction with EIN_085620.

## DISCUSSION

Stage conversion in the life cycle of *Entamoeba* is a critical biological process for the parasite’s survival outside the host and for disease transmission. In order to determine the transcriptional regulators that control the early aspects of *Entamoeba* development, we identified proteins that bind a conserved promoter motif (TCACTTTC) in the promoters of genes upregulated at 8 h of encystation. Identification of transcriptional regulators at this early time point is crucial because this is when a series of transcriptomic and phenotypic changes are triggered, establishing the groundwork for complete encystation. This work identifies the earliest regulators of development in *Entamoeba*, when only clumping and aggregation are noted, but before any structural changes are seen in the parasites.

Analysis by LC-MS allowed the identification of two nuclear proteins, EIN_066100 and EIN_085620, involved in the growth and differentiation of *E. invadens*. EIN_066100 and EIN_085620 genes have a low expression level in trophozoites but are upregulated during early encystation, indicating that their expression itself may be subject to regulation by encystation signals. Furthermore, it is noteworthy that both EIN_066100 and EIN_085620 are localized within the nucleus during the trophozoite stage and around the nucleus during the cyst stage, which is an important requirement for their potential role as transcription factors.

One of the most interesting findings in our study is that based on the function of their homologs in other systems, EIN_066100 and EIN_085620 are proteins associated with RNA processes. The homolog of EIN_066100 in *Saccharomyces cerevisiae,* MAK16, has been implicated both in cell cycle progression (22) and biogenesis of 60S ribosomal subunits (23), whereas in *Schistosoma mansoni,* Mak16 associates with pre-ribosomal precursor complexes (25). Previously, a Mak16 gene in *Entamoeba* was identified in isolates obtained from symptomatic patients and hamsters with liver damage, suggesting an association of Mak16 expression with the development of amebic liver abscess (29). Our results suggest a new role of this gene in amoeba biology, coding for a protein that binds DNA and regulates *Entamoeba* proliferation and encystation. Further investigations could help elucidate whether EIN_066100 is also associated with ribosomal functions in *Entamoeba*. EIN_085620 homologs have been shown to have variable functions. In mice, the activator of basal transcription 1 (mABT1) is a nuclear protein that binds TATA box binding protein (TBP) and DNA and enhances Pol II-directed basal transcription (27), whereas in yeast, Esf2 protein interacts directly with the RNA helicase Dbp8 to stimulate ATP hydrolysis (26).

Recently, an *in silico* analysis, to infer the gene regulatory networks and identify families of transcription factors in different *Entamoeba* species, predicted 297 potential transcription factors in *E. invadens* most of which were associated with the RRM_1 family (PF00076) (30). Nevertheless, no protein containing this domain has been functionally characterized in *Entamoeba*. The RRM domain in EIN_085620 sequence thus supports its potential role as a transcription factor. The ability to bind DNA, RNA, and protein is a general property of many transcription factors and is fundamental to their gene regulatory function (31). Interestingly, other proteins associated with some ribosomal functions like eukaryotic translation initiation factor two alpha kinases (eIF2α) have been involved in controlling parasite stress response, stage conversion, and virulence (32). These findings indicated that these proteins perform distinct roles within a cell and these additional functions may contribute to the complexity and adaptability of cellular and molecular processes.

One of the limitations of our study is that we were unable to silence the expression of EIN_066100 and EIN_085620, suggesting that downregulation of these genes might be essential to the parasite. As an alternative approach to identify the effects of loss of function of these proteins, we attempted to express truncated proteins with deletions at either the N-terminal or C-terminal regions. However, only the mutants EIN_066100∆C and EIN_066100∆N were successfully expressed.

It has been described that deletions in proteins either disrupt or enhance interactions with other proteins. Such alterations can lead to the formation of new protein complexes or the loss of regulatory interactions, ultimately resulting in changes in cellular processes (33). The phenotypic outcomes observed in parasites expressing EIN_066100 mutants indicated that disruption of the structure of this protein affects its subcellular localization and might be modifying their interactions with other proteins. Deletion of the N-terminal region of EIN_066100 caused protein mis-localization and negative effects on cellular processes such as proliferation, encystation, and lack of interaction with EIN_085620. In contrast, deletion of the C-terminal region led to a gain-of-function, enhancing encystation. This suggests that the C-terminal region might normally adopt a structure that regulates the protein’s activity. Further investigations could uncover interaction interfaces within the C-terminal region that cause unpredicted functional conformational changes.

**Fig 5:**
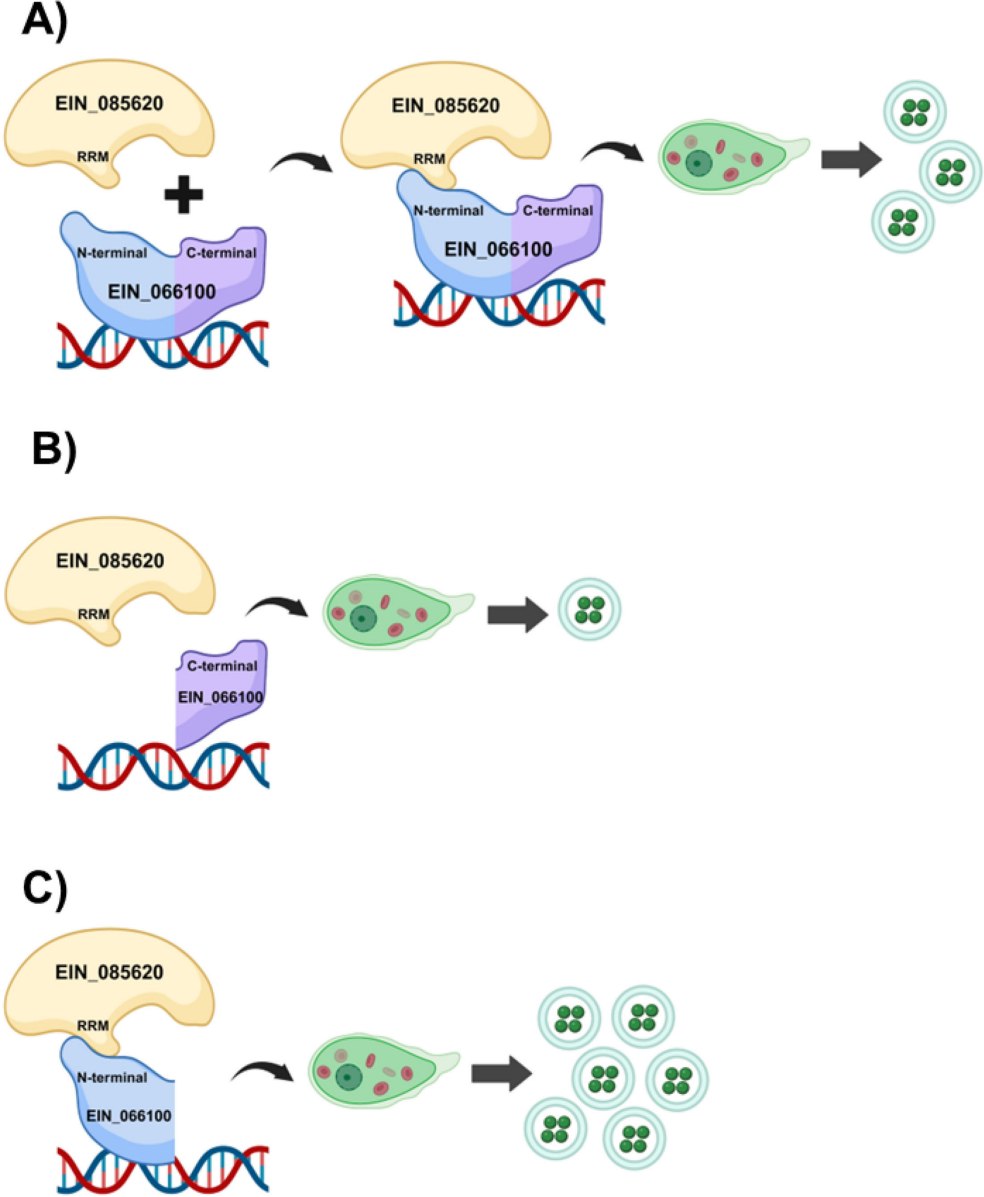
Proposed model for the role of EIN_066100 and EIN_085620 in *Entamoeba* encystation. (**A**) EIN_066100 and EIN_085620 interact via EIN_085620 RRM domain and the N-terminal region of EIN_066100. This interaction suggests that EIN_085620 acts as a rate-limiting factor of EIN_066100 and promotes encystation. (**B**) Deletion of the N-terminal region of EIN_066100 caused a lack of interaction with EIN_085620 and a reduction in the number of cysts, indicating that this region is essential for initiating the encystation cascade. (**C**) Deletion of C-terminal EIN_066100 enhances encystation, raising the possibility that the C-terminal region of EIN_066100 functions to control or limit interaction efficiency and thus controls developmental efficiency.

Transcription factors orchestrate gene expression either by binding directly to DNA sequences or indirectly by interacting with other proteins like coactivators or corepressors (34). Based on our findings, we propose that EIN_066100 and EIN_085620 are part of the same protein complex that recognizes the DNA motif. However, we propose that EIN_066100 interacts directly with DNA, whereas EIN_085620 might have an indirect role by interacting through its RRM domain with the N-terminal region of EIN_066100. Moreover, our GST pull down assays showed that when EIN_085620 was used as bait, the interaction with EIN_066100 was stronger than when EIN_066100 was used as bait, suggesting that EIN_085620 might be acting like a rate-limiting factor for the interaction. In terms of phenotypic effects, overexpression of EIN_066100 WT is controlling the proliferation, whereas EIN_085620 WT is promoting the encystation, suggesting a coordinated role in regulating different cellular processes associated with *Entamoeba* development. Taking together, we propose a model for the role of EIN_066100 and EIN_085620 in *Entamoeba* encystation (Fig. 5). The identification of new transcriptional regulators in *Entamoeba* addresses an important aspect of parasite biology.

## MATERIALS AND METHODS

### Parasite culture, *in vitro* encystation, and growth kinetics

*E. invadens* trophozoites strain IP-1 was axenically maintained in LYI-S-2 media (35, 36). To induce *in vitro* encystation, *E. invadens* trophozoites were harvested in the late logarithmic phase and incubated in 47% LYI-LG (low glucose) supplemented with 7% adult bovine serum at a final concentration of 5 × 10^5^ cells/mL (37). Cells were collected at different time points 0, 8, 24, and 72 h after exposure to the encystation medium according to each experiment. To measure encystation efficiency, total encysting cells at 72 h were counted using a hemocytometer before and after treatment with 0.1% sarkosyl.

For growth curves, trophozoites in the mid-log phase were harvested and seeded at 40,000 cells per tube in 9 mL of LYI-S-2 media. The number of cells was counted on days 1, 3, 5, 7, and 9. The experiments were repeated three times with two replicates for each sample.

### Bioinformatics analysis to identify consensus DNA promoter motifs

In total, 500 nt of the upstream promoter regions of 502 genes upregulated at 8 h post-encystation were extracted from https://amoebadb.org/amoeba/app/ and analyzed to identify DNA motifs as described earlier (21, 38) using the MEME suite (39). MEME analysis was performed with the command line: -dna -mod zoops -nmotifs 20 -minw 5 -maxw 8 -minsites 5. FIMO program was used to determine the total number of occurrences of each motif in the promoter sequence databases. The hypergeometric distribution was used to determine the significance of enrichment for each motif identified. Motifs with *P*-value of less than 0.001 were determined to be enriched significantly within the promoters of the 8 h cyst-specific genes. Sequence logos were generated automatically by MEME program.

### Electrophoretic mobility shift assays

EMSA assays were performed as previously described (38) with minor changes. The oligonucleotides used in EMSA are listed in Table S3. Binding reaction was set in a total volume of 20 µL, which included 2 µL 10× EMSA-binding buffer (10 mM Tris-HCl [pH 7.9], 50 mM NaCl, 1 mM EDTA, 3% glycerol, 0.05% BSA, and 0.05 mg of bromophenol blue), 6 µg of nuclear extract from trophozoites or 8 h or 24 h cysts, 1 µg/µL of poly(dI-dC), and 50 fmol of labeled probe. For supershift assays, 6 µg of nuclear extracts was preincubated for 3 h at 4°C in 20 mM HEPES buffer with 2 µg of each antibody: rabbit anti-Myc-tag (71D10) and rabbit anti-beta actin (ab227387). The binding reaction mix was incubated for 30 min at room temperature, and samples were loaded onto a 6% non-denaturing polyacrylamide gel and run for 2.5 h. The gel was fixed, dried, and exposed to a phosphor screen. Gels were imaged using Personal Molecular Imager (PMI) System with Quantity One software, Bio-Rad.

### Mass spectrometry identification of M9-binding proteins

To identify the M9-binding protein, we performed LC-MS excising the shifted band from EMSA gel. EMSA was performed by using M9-WT, M9 Core probes with 8 h cyst nuclear proteins. Gel slices were excised from the unlabeled lanes of M9-WT, M9 Core from two biological experiments. In one experiment, EMSA was performed by using the ERM-BP probe, which served as an extra negative control. Gel slices were reduced with 10 mM of dithiothreitol (DTT) and alkylated with acrylamide followed by digestion with trypsin/LysC mix (Promega). Samples were quenched and dried prior to their reconstitution. Mass spectrometry experiments were performed using an Orbitrap Eclipse Tribrid mass spectrometer (Thermo Scientific). The RAW data were analyzed and visualized using Byonic v4.1.5 software to identify peptides and infer proteins. An *in silico* peptide library was generated and proteins were held to a false discovery rate of 1% using a standard reverse-decoy technique (40).

### Plasmid construction

For overexpression in *E. invadens*, the full-length coding region of EIN_066100 (798 bp), EIN_085620 (678 bp), and mutants EIN_066100∆C (426 bp) and EIN_066100∆N (381 bp) were amplified by PCR and cloned into the pEiCK-Myc plasmid by using AvrII and SacII restriction sites, and constructions were confirmed by sequencing. For the generation of stable transgenic cell lines, parasites were transfected with plasmid DNA by electroporation (41). The stable cell lines were maintained at a G418 concentration of 80 µg/mL.

For bacterial expression of recombinant proteins, ORF of EIN_066100, EIN_085620, and EIN_085620 mutants were amplified and cloned into the pGEX-4T-1 vector with GST tag on N-terminal using BamHI and NotI restriction sites and into the pET-28b vector with a 6xHis-tag on N-terminal using HindIII and NotI restriction sites. Resultant plasmids were transformed in *Escherichia coli* BL21(DE3) and confirmed by sequencing. The primers for cloning used in this study are listed in Table S3.

### RNA extraction, cDNA synthesis, and RT-PCR

Total RNA was isolated from trophozoites and cysts using TRIzol method (Life Technologies). Two micrograms of total RNA was treated with ezDNase enzyme (Invitrogen) at 37°C for 20 min, and cDNA synthesis was performed using SuperScript IV VILO Master Mix (Invitrogen) according to the manufacturer’s instructions. The resultant cDNA was diluted 1:10 in H_2_O nuclease-free solution, and 2 µL of diluted cDNA was used to perform subsequent PCRs (25 µL total volume). The number of PCR cycles was set at 25, and PCR products were run on a 2% agarose gel and stained with ethidium bromide. The primers used for RT-PCR are listed in Table S3.

### Expression and purification of recombinant proteins

GST-tagged proteins were expressed in *E. coli* BL21(DE3) and purified using glutathione beads (Thermo Scientific). Briefly, cells cultured overnight were diluted 1:50 into 100 mL LB and incubated at 37°C until the cell density reached an optical density at 600 nm (OD600) of 0.7–0.8. Proteins were induced under different IPTG concentrations and temperatures: GST – EIN_066100-FL was induced with 0.25 mM IPTG for 4 h at room temperature; GST-EIN_085620-FL and GST-EIN_085620∆N were induced with 0.5 mM IPTG for 4 h at 37°C; and GST-EIN_085620∆C, GST-EIN_085620 1–45, and GST-EIN_085620-RRM were induced with 0.1 mM IPTG for 4 h at room temperature. After induction, cells were collected, and pellets from GST-EIN_066100-FL, GST-EIN_085620-FL, and GST-EIN_085620∆N were resuspended in GST-binding buffer (25 mM Tris-HCl pH 7.5, 150 mM NaCl, 10 mM MgCl_2_) plus 0.2% Triton X-100, 5 mM DTT, 1 mM PMSF, and 1X HALT (protease inhibitor cocktail), whereas GST-EIN_085620∆C, GST-EIN_085620 1–45, and GST-EIN_085620-RRM were resuspended in STE-binding buffer (10 mM Tris-HCl [pH 8.0], 150 mM NaCl, 1 mM EDTA) plus 1.5% sarkosyl, 5 mM DTT, 1 mM PMSF, and 1× HALT. Samples were incubated on ice for 10 min and then lysed by sonication. Samples were centrifugated at maximum speed for 10 min at 4°C, and the supernatants were collected. For purification, glutathione beads were equilibrated with GST-binding buffer or STE-binding buffer, and supernatants from samples sonicated with GST-binding buffer were incubated directly with beads, whereas samples sonicated with STE-binding buffer were diluted 1:10 with STE-binding buffer, and Triton X-100 was added to a 2% final concentration. The samples were rotated overnight at 4°C. The beads were spun down at 2,000 rpm for 2 min and washed three times with GST-wash buffer (50 mM Tris-HCl [pH 7.5], 500 mM NaCl, 1 mM PMSF, and 0.1% Triton X-100). Bound proteins were eluted with elution buffer (20 mM reduced glutathione, 50 mM Tris-HCl [pH 8.0], 10 mM MgCl_2_, 1 mM PMSF, 1× HALT). Purified protein samples were dialyzed overnight at 4°C in dialysis buffer (5 mM HEPES [pH 7.9], 1 mM EDTA, 1 mM DTT, 0.2 mM PMSF, 10% glycerol) with two changes to remove glutathione.

His-tagged proteins were expressed in *E. coli* BL21(DE3) and purified using HisPur Ni-NTA Resin (Thermo Fisher Scientific). His-EIN_066100-FL was induced with 0.25 mM IPTG for 4 h at room temperature. Cells were pelleted and resuspended in His-binding buffer (50 mM Tris-HCl [pH 8.0], 100 mM NaCl, 10 mM imidazole, 0.1 mM EDTA [pH 8.0], 5% glycerol) plus 0.25% NP40, 3 mM β-mercaptoethanol, 1 mM PMSF, and 1× HALT. The sample was incubated on ice for 10 min and then lysed by sonication. The sample was centrifugated at maximum speed for 10 min at 4°C, and the supernatant was collected. Ni-NTA beads were equilibrated with His-binding buffer, and the supernatant was added and rotated overnight at 4°C. The beads were spun down at 2,000 rpm for 2 min and then washed with His-wash buffer (50 mM Tris-HCl [pH 8.0], 300 mM NaCl, 20 mM imidazole, 0.1 mM EDTA, 5% glycerol, 3 mM β-mercaptoethanol, 1 mM PMSF, and 0.05% NP40). Bound proteins were eluted with elution buffer (50 mM Tris-HCl [pH 8.0], 50 mM NaCl, 300 mM imidazole, 100 mM KCl, 0.1 mM EDTA [pH 8.0], 5% glycerol, 1 mM PMSF, and 1× HALT). Finally, 1 mM EDTA and 1 mM DTT were added to the eluted sample and then dialyzed overnight at 4°C in dialysis buffer (25 mM Tris-HCl [pH 8.0], 50 mM NaCl, 100 mM KCl, 0.2 mM EDTA, 5% glycerol, 1 mM DTT, and 0.2 mM PMSF). Total protein was quantified by the Bradford method and checked by SDS-PAGE.

### Immunofluorescence assay

Trophozoites attached on cover slides and cysts in suspension were processed according to the protocol as previously reported (11). Samples were incubated with primary anti-Myc mouse antibody (Cell Signaling 9B11) at 1:250 dilution overnight at 4°C. Alexa Fluor-488 Goat Anti-Mouse IgG antibody (Invitrogen A-11001) was used as a secondary antibody at a 1:1,000 dilution. The samples were mounted using DAPI Fluoromount-G (Southern Biotech) and observed using Olympus BX60 fluorescence microscope. Images were analyzed using ImageJ software.

### Parasite cell lysate

Two T25 flasks of confluent parasite culture were resuspended in 2 mL IP buffer (20 mM Tris-HCl [pH 7.5], 1 mM MgCl_2_, 50 mM NaCl, 10% glycerol) supplemented with 0.5% NP-40, 1 mM DTT, 1 mM PMSF, 1 mM E64, and 2× HALT. Samples were rotated at 4°C for 45 min, the cell lysate was centrifuged at maximum speed for 30 min at 4°C, and the supernatant was recovered. Amoebic lysate protein levels were quantified by Bradford method and used to perform western blot analysis and GST pull down assays.

### Western blot analysis

Proteins were separated on 12% polyacrylamide SDS-PAGE and transferred to a polyvinylidene fluoride (PVDF) membrane. The membranes were blocked with PBS containing 1% casein (Bio-Rad) and incubated with mouse anti-Myc-tag (Cell Signaling 9B11) (1:2,000) overnight at 4°C. The membranes were rinsed with PBS-Tween 0.1%, and anti-mouse IgG HRP-linked antibody (Cell Signaling 7076) (1:10,000) was used as the secondary antibody. The signal was detected with ECL (GE Healthcare), and the film was developed by a film processor (Konica).

### GST pull down assay

All GST-tagged proteins served as “bait” for capturing Myc-tagged “prey” binding partners. For each sample, 25 µL of equilibrated glutathione beads was used, and 250 µg bacteria lysate was added to immobilize bait proteins. The sample mixture was rotated at 4°C overnight, and the beads were washed three times with GST-wash buffer. Afterward, 500 µg amebic lysates containing prey proteins were added to the beads. The samples were rotated at 4°C for 1 h. The bound complexes were washed with IP-wash buffer (IP buffer supplemented with 0.1% Tween 20, 0.1% NP40, 0.5 mM DTT). The beads were resuspended with 50 µL 2× Laemmli sample buffer, boiled for 5 min, and separated by SDS-PAGE and stained with Coomassie Brilliant Blue R-250 (Biorad).

### Statistical analysis

All data were processed and analyzed using GraphPad Prism 8.0.1 software. For multiple group comparisons, either one-way or two-way ANOVA was used, depending on the experimental design. The results are expressed as the mean ± standard deviation (SD), derived from at least three independent biological experiments, each conducted with two technical replicates.

## SUPPLEMENTAL MATERIAL

Fig. S1 mbio.02250-24-s0001.tiff Motifs enriched in the promoters of genes that upregulated at 8 h of encystation.

Fig. S2 mbio.02250-24-s0002.tiff Summary of MS results to identify M9-binding proteins.

Fig. S3 mbio.02250-24-s0003.tiff Sequence alignment between EIN_066100 and EIN_085620 and their homologs in *E. histolytica*.

Fig. S4 mbio.02250-24-s0004.tiff Bacterial expression of recombinant proteins utilized for GST pulldown assays.

Legends mbio.02250-24-s0005.docx Legends for supplemental figures.

Table S1 mbio.02250-24-s0006.xlsx Genes upregulated at 8 h post-encystation.

Table S2 mbio.02250-24-s0007.xlsx Proteins identified by LC-MS.

Table S3 mbio.02250-24-s0008.xlsx Oligonucleotides used in this study.

ASM does not own the copyrights to Supplemental Material that may be linked to, or accessed through, an article. The authors have granted ASM a non-exclusive, world-wide license to publish the Supplemental Material files. Please contact the corresponding author directly for reuse.
