# Supplementary figures and images for: Identification of two transcription factors that work coordinately to regulate early development in Entamoeba
Source: mBio. 2024 Nov 14;15(12):e02250-24. doi: 10.1128/mbio.02250-24 (PMC11633172; doi:10.1128/mbio.02250-24)

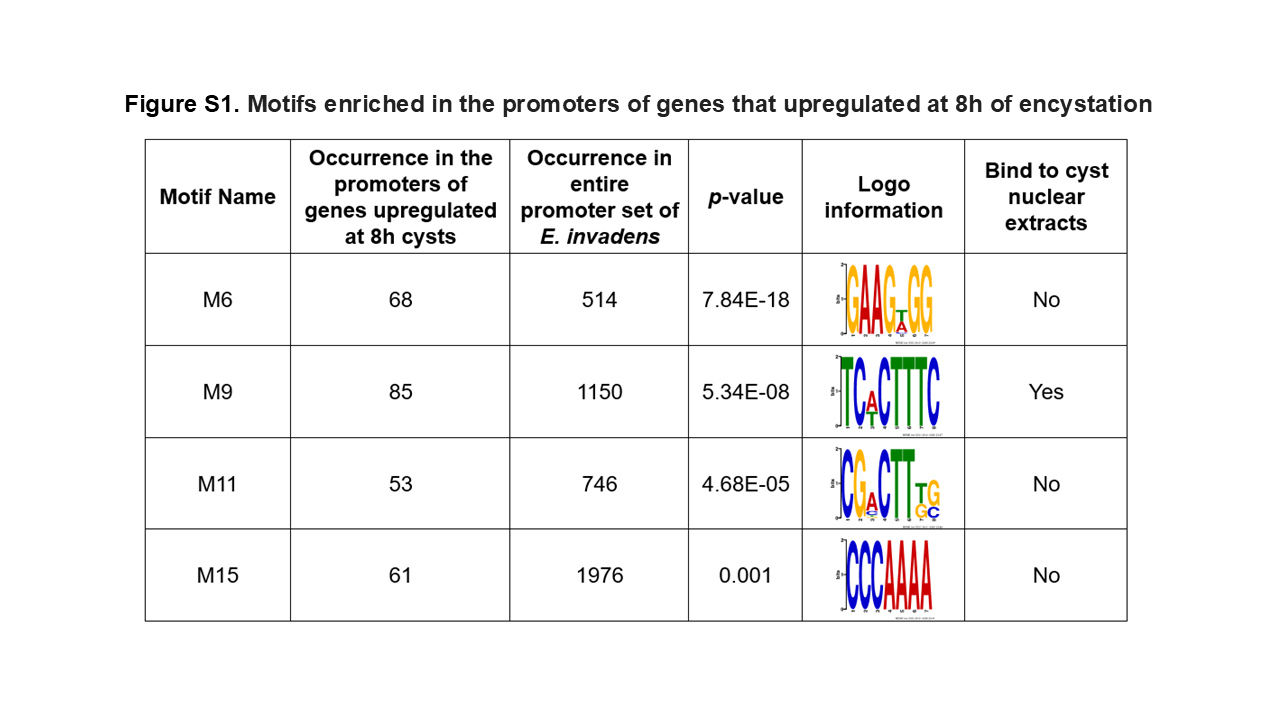

Supplement: Fig. S1 — Motifs enriched in the promoters of genes that upregulated at 8 h of encystation. [file mbio.02250-24-s0001.tiff]

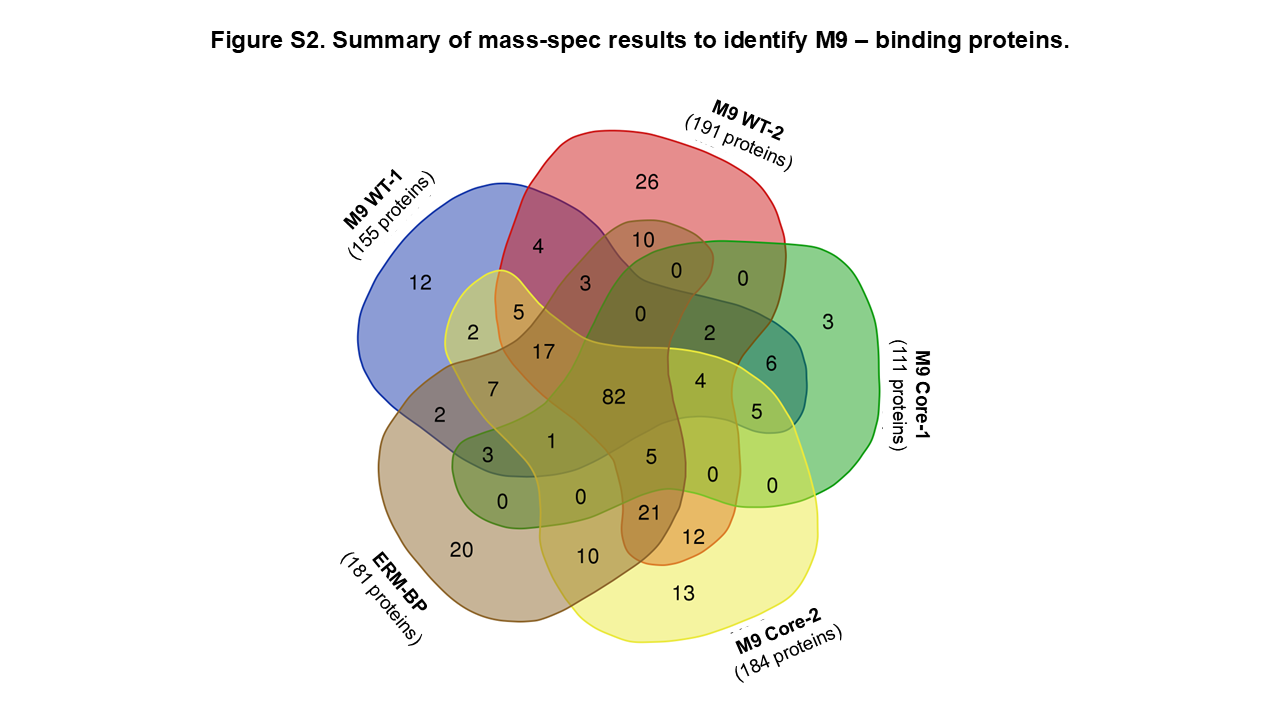

Supplement: Fig. S2 — Summary of MS results to identify M9-binding proteins. [file mbio.02250-24-s0002.tiff]

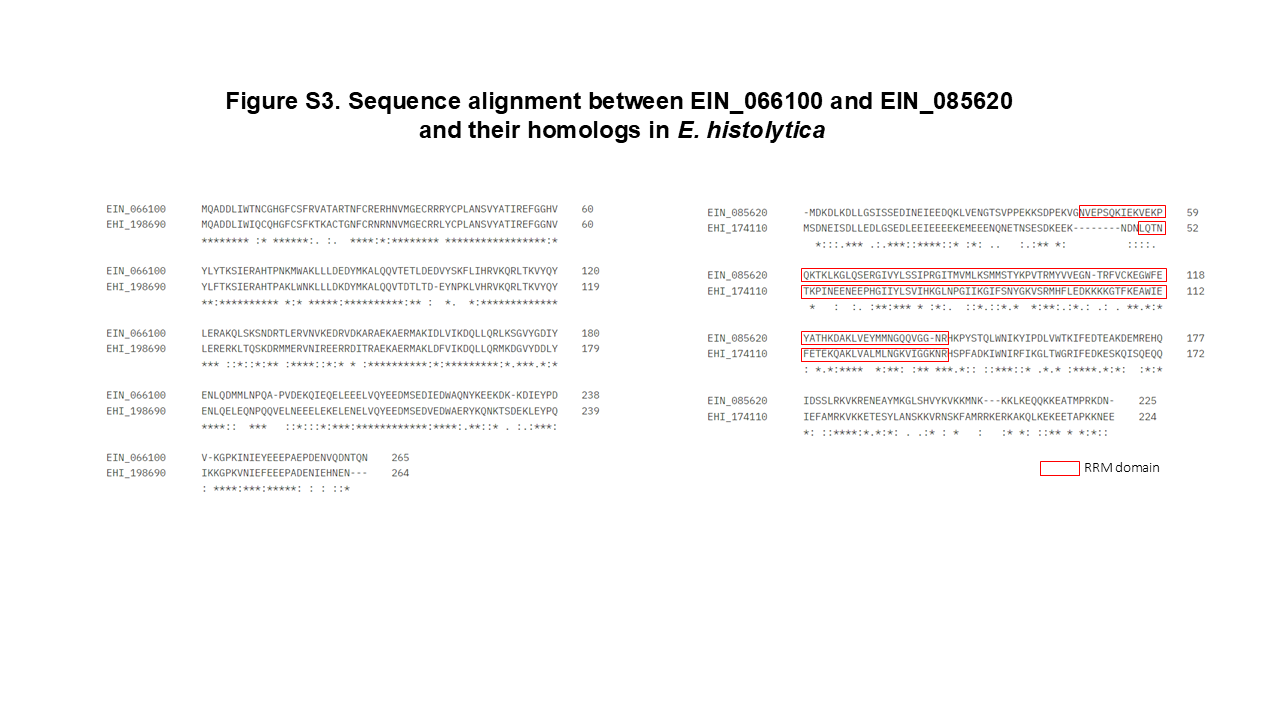

Supplement: Fig. S3 — Sequence alignment between EIN_066100 and EIN_085620 and their homologs in E. histolytica. [file mbio.02250-24-s0003.tiff]

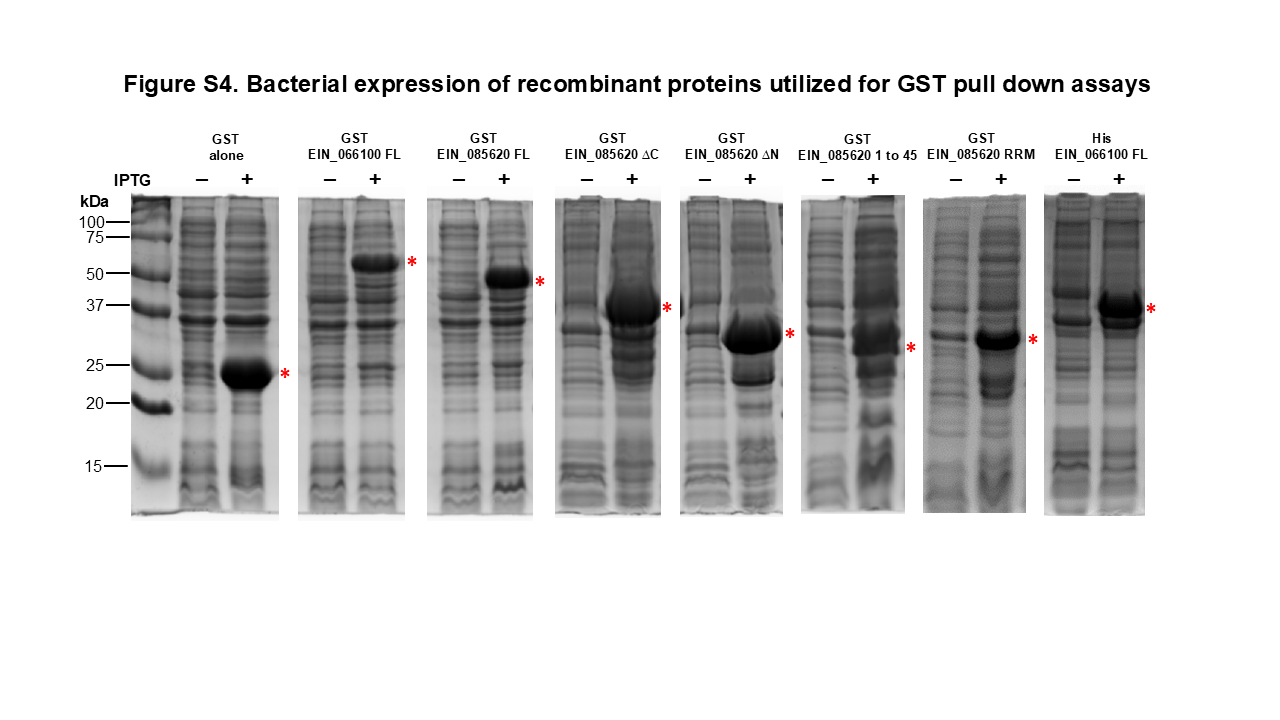

Supplement: Fig. S4 — Bacterial expression of recombinant proteins utilized for GST pulldown assays. [file mbio.02250-24-s0004.tiff]
