# Supplementary material for: Identification of two transcription factors that work coordinately to regulate early development in Entamoeba
Source: mBio. 2024 Nov 14;15(12):e02250-24. doi: 10.1128/mbio.02250-24 (PMC11633172; doi:10.1128/mbio.02250-24)
Supplement: Legends — for supplemental figures. [file mbio.02250-24-s0005.docx]

**Figure S1. Motifs enriched in the promoters of genes that are upregulated at 8h post-encystation.** Four motifs (M6, M9, M11 and M15) were significantly enriched among the promoters of 502 genes upregulated at 8h post-encystation compared to all promoter database in *E. invadens*. Motif name, number of occurrences in entire promoter set of 8h cyst-specific genes, number of occurrences in all promoters, p-values, motif information logos and screening results of the motifs by electrophoretic mobility shift assay (EMSA) are listed.

**Figure S2. Summary of mass-spec results to identify M9-binding proteins.** LC-MS data analysis from two independent experiments is shown. The number of proteins identified in each sample (M9 WT-1 M9 WT-2, M9 Core-1, M9 Core-2 and ERM-BP are indicated in parenthesis) were compared. Only four proteins met the criteria of being exclusively identified in M9-WT compared with negative controls.

**Figure S3. Sequence alignment between EIN_066100 and EIN_085620 and their homologs in *E. histolytica.*** Protein sequence alignments between *E. invadens* EIN_066100 and *E. histolytica* EHI_198690A (left side) and *E. invadens* EIN_085620 and *E. histolytica* EHI_174110A (right side) were performed by using Clustal-Omega. The red box indicates the RRM domain.

**Figure S4. Bacterial expression of recombinant proteins utilized for GST pull down assays.** GST-fusion proteins (GST alone, GST-EIN_066100-FL, GST-EIN_085620-FL, GST-EIN_085620∆C, GST-EIN_085620∆N, GST-EIN_085620 1 to 45 and GST-EIN_085620 RRM) and His-tag protein (His-EIN_066100-FL) were expressed upon IPTG induction. The cell pellets before and after IPTG induction were lysed using 1x Laemmli buffer in 1X PBS and SDS-PAGE gel was stained by Coomassie Blue G-250. Red asterisk is pointing the induced band for each protein which match to the predicted protein size: GST alone 26 kDa, GST-EIN_0666100-FL 57 kDa, GST-EIN_085620-FL 52 kDa, GST-EIN_085620∆C 42 kDa, GST-EIN_085620∆N 36 kDa, GST-EIN_085620 1 to 45 31kDa, GST-EIN_085620 RRM 37 kDa, His-EIN_066100-FL 36 kDa.
